# Small Ribosomal Protein Subunit S7 Suppresses Ovarian Tumorigenesis through Regulation of the PI3K/AKT and MAPK Pathways

**DOI:** 10.1371/journal.pone.0079117

**Published:** 2013-11-11

**Authors:** Ziliang Wang, Jing Hou, Lili Lu, Zihao Qi, Jianmin Sun, Wen Gao, Jiao Meng, Yan Wang, Huizhen Sun, Hongyu Gu, Yuhu Xin, Xiaomao Guo, Gong Yang

**Affiliations:** 1 Cancer Institute, Fudan University Shanghai Cancer Center, Department of Oncology, Shanghai Medical College, Fudan University, Shanghai, China; 2 Department of Radiation Oncology, Fudan University Shanghai Cancer Center, Department of Oncology, Shanghai Medical College, Fudan University, Shanghai, China; 3 Department of Gynecological Oncology, Fudan University Shanghai Cancer Center, Department of Oncology, Shanghai Medical College, Fudan University, Shanghai, China; 4 Life and Environment Science College, Shanghai Normal University, Shanghai, China; Institute of Hepatology - Birkbeck, University of London, United Kingdom

## Abstract

Small ribosomal protein subunit S7 (RPS7) has been reported to be associated with various malignancies, but the role of RPS7 in ovarian cancer remains unclear. In this study, we found that silencing of RPS7 by a specific shRNA promoted ovarian cancer cell proliferation, accelerated cell cycle progression, and slightly reduced cell apoptosis and response to cisplatin treatment. Knockdown of RPS7 resulted in increased expression of P85α, P110α, and AKT2. Although the basal levels of ERK1/2, MEK1/2, and P38 were inconsistently altered in ovarian cancer cells, the phosphorylated forms of MEK1/2 (Ser217/221), ERK1/2 (Thr202/Tyr204), JNK1/2 (Thr183/Tyr185), and P38 (Thr180/Tyr182) were consistently reduced after RPS7 was silenced. Both the in vitro anchorage-independent colony formation and in vivo animal tumor formation capability of cells were enhanced after RPS7 was depleted. We also showed that silencing of RPS7 enhanced ovarian cancer cell migration and invasion. In sum, our results suggest that RPS7 suppresses ovarian tumorigenesis and metastasis through PI3K/AKT and MAPK signal pathways. Thus, RPS7 may be used as a potential marker for diagnosis and treatment of ovarian cancer.

## Introduction

Ovarian cancer is the most lethal gynecological malignancy worldwide [1]. Ribosomal proteins (RPs) play essential roles in the formation of a fully functional ribosome, which is responsible for protein synthesis in both prokaryotic and eukaryotic cells. Over the past decades, more and more RPs have been identified to exert diverse functions in addition to protein synthesis [2,3]. Some studies have shown that the dysfunction of ribosomal proteins results in dysregulation of protein translation, leading to the development of many cancers [4,5]. However, the underlying mechanisms are largely unknown. 

Some studies have shown that RP genes are oncogenes in human tissues. Ever since RPS19 was found to have a close relationship with progression and differentiation of colon carcinoma 20 years ago [6], RPs have been reported to have important roles in many cancers. For example, silencing of ribosomal proteins L26 and L29 by small interfering RNAs (siRNAs) inhibited the proliferation of human pancreatic cancer cells [7]. Knockdown of the ribosomal protein RPL19 by siRNA abrogated the aggressive phenotype of human prostate cancer [8]. The ribosomal protein L6 promoted cell cycle progression through up-regulating cyclin E in gastric cancer cells [9]. Overexpression of the ribosomal protein L15 was associated with cell proliferation in esophageal cancer [10]. Overexpression of RPL36a enhanced cellular proliferation in hepatocellular carcinoma [11]. Another study demonstrated that RPS7 may block the function of p53/MDM2 and abrogate the Ling Zhi-8-induced lung cancer cell proliferative advantage [12]. In our previous work, we used zebrafish as a model and found that at the early stage of embryonic development, RPS7 suppressed cell apoptosis and cell cycle progression through dysregulation of p53 [13,14], indicating that RPS7 might be an important factor in human cancers. However, in 2007, RPS7 was also identified to activate p53 and MDM2, which subsequently promoted cellular apoptosis and inhibited cell proliferation [15]. Thus, the regulatory function of RPs in tumorigenesis is controversial and still needs further investigations.

To elucidate the biological function of RPS7 in ovarian cancer, we silenced the expression of RPS7 by specific shRNA in ovarian cancer cell lines and tested the effects of RPS7 on cell proliferation, apoptosis, cell cycle, migration, invasion, and tumorigenesis. Our results demonstrate that RPS7 suppresses ovarian tumorigenesis and metastasis through dysregulation of PI3K/AKT and MAPK signal pathways.

## Materials and Methods

### Ethics statement

All mouse experiments were approved by the Institutional Animal Care and Use Committee of Fudan University Shanghai Cancer Center, and performed following the institutional guidelines. 

### Cell lines and cell culture

Human epithelial ovarian cancer cell lines OVCA433, OVCA429, OVCA420, SKOV3, and retroviral packaging cells (Phoenix amphotropic cells) were purchased from American Type Culture Collection (Manassas, VA). Immortalized human ovarian surface epithelial cell line T29 was derived from the normal ovarian surface epithelial cell line IOSE29, which was described elsewhere [16,17]. OVCA433, OVCA429, T29 and phoenix cells were maintained in DMEM medium supplemented with 10% fetal bovine serum, 2 mM l-glutamine, nonessential amino acids (1%), 1 mM sodium pyruvate, penicillin (100 units/mL), and streptomycin (100 µg/mL). SKOV3 and OVCA420 cells were maintained in RPMI 1640 medium containing 10% fetal bovine serum, 2 mM l-glutamine, penicillin (100 units/mL), and streptomycin (100 µg/mL).

### Cisplatin treatment

Cisplatin was purchased from QiLu pharmaceutical company (Shanghai, China). Stock concentration of cisplatin was 5mg/ml and the concentration used to treat ovarian cancer lines was 2.5 µg/mL. The apoptosis of cells was detected by Nicoletti assay [18].

### Generation and retroviral delivery of small hairpin RNA (shRNA) against RPS7 mRNA

The DNA oligonucleotides used to generate shRNA against the open reading frame of RPS7 mRNA (positions 167–189) were 5-TCGGAAAGCTATCATAATCTTTa-3. pBabe/U6/shPRS7 was generated according to the previously reported method [19,20]. The control vector was similarly constructed by directly inserting oligonucleotides encoding small hairpin RNA against green fluorescence protein mRNA (shGFP) into pBabe/U6/puromycin [19,21].

Retroviruses expressing RPS7 shRNA or GFP shRNA were produced by transfection of pBabe/U6/shRPS7 or pBabe/U6/shGFP into phoenix amphotropic cells and used to infect target cells (SKOV3 , OVCA429, and OVCA433) by using a method described before [19]. Briefly, cells were infected twice for a total of 6 days (3 days for each infection) and the positive clones were selected with puromycin (200 ng/mL) for 10–14 days to establish stable cell lines expressing shRPS7 or shGFP. The resulting cells were used for following experiments without addition of puromycin.

### Cell proliferation

Cells were detached by using trypsinization and washed twice with PBS. 2×10^3^ cells per well were seeded in 96-well culture plates (Corning Inc., Corning, NY) in 100 μl medium and cultured for 2, 4, 6, 8, 10 days. Cell growth was detected using Cell Counting Kit-8(CCK-8) (Dojindo Laboratories, Kumamoto, Japan) according to the manufacturer’s instructions. Absorbance at 450 nm was measured with a microplate reader. The assay was independently repeated three times.

### Cell invasion and migration

To test cell invasion, we used a high throughput screening multi-well insert 24-well two-chamber plate (BD Biosciences, San Jose, CA), with an 8-µm (pore size) polycarbonate filter between chambers. 2.5 × 10^3^ cells of SKOV3-shRSP7, OVCA429-shRPS7 or OVCA433-shRSP7 and their corresponding controls expressing shGFP were added in upper chamber and allowed to invade at 37°C for 24 hours toward a lower reservoir containing medium plus fibronectin (20 µg/mL). The cells were then fixed in 100% methanol for 30 minutes and stained with Giemsa solution for 10 minutes. The invasive cells were counted as those passed through the membrane separating the chamber. All cells were counted at ×200 magnification under a microscope. The assay was repeated three times with duplicate.

To examine cell migration, cells were incubated in 6-well plate over-night to yield monolayer confluence for scratch assay. Scratches were made using a pipette tip and photographed immediately (time 0) and 24 hours’ later. The distance migrated by the cell monolayer to close the scratch area during the time period was measured. Results were analyzed as migration index, which was the ratio of the cell migration distance at 24h to that at 0h.The assay was carried out in triplicate and repeated three times.

### Anchorage-independent colony formation

To detect anchorage-independent colony growth, soft agar assay was performed with cells stably expressing either shRPS7 or shGFP according to the previously published method [19,20]. Briefly, 5 × 10^4^ cells were suspended in 2 mL of medium with 0.35% agarose (Life Technologies), and the suspension was placed on top of 5 mL of solidified 0.7% agarose. Triplicate cultures of each cell type were maintained for 14-28 days at 37°C in a 5% CO2 atmosphere, and fresh medium was fed every 7 days. The number of colonies > 50 µm (~100 cells) in diameter in each dish was counted at 14 to 20 days. The assay was repeated three times in duplicate.

### Cell cycle and apoptosis

Cells (1–2 × 10^6^) were fixed with 4 mL of cold 75% ethanol at 4°C for a minimum of 4 hours and stained with 200 µL of propidium iodide (50 µL/mL; Sigma-Aldrich) and 20 µL of RNase (1 mg/mL; Sigma-Aldrich) in a 37°C water bath for 15 to 20 minutes. Cell cycles were determined by FACStation (BD Biosciences) and analyzed by using CellQuest software and a published method [19]. The assay was repeated three times in duplicate. This method was also used to detect the percentage of sub-G1 cells, which represents cellular apoptosis after cells were treated with or without cisplatin.

To detect apoptosis, 1 × 10^5^ cells were stained with Annexin V and propidium iodide according to the instruction of the Annexin V–fluorescence apoptosis detection kit I (BD Biosciences PharMingen), and to analysis with a FAC Station equipped with CellQuest software. The percentage of apoptotic cells was calculated in terms of peaks (M2) in the histogram, representing an early apoptotic population (Annexin V+/PI−) among the total cells analyzed. The experiment was done in duplicate and repeated three times. 

### Real-time PCR

Total RNA from 2 × 10^6^ cells for each cell line was isolated by using Trizol reagent (Invitrogen, Carlsbad, CA). All RNAs were then reversely transcribed into cDNAs that were suitable for real-time PCR analysis using the ExScript RT-PCR kit (TaKaRa, Japan). To synthesize cDNA, 0.5 mM deoxynucleoside triphosphate, 50 pmol random hexamers, 50 U ExScript reverse transcriptase (200 U/μl), 10 U RNase inhibitor, 500 ng total RNA, and 1 × reaction buffer were mixed in each reaction tube (10μl per reaction) and then incubated at 42°C for 15 min, followed by a 2-min incubation at 95°C to inactivate the ExScript reverse transcriptase. Oligonucleotide primers for RPS7 were 5’-GTCGTCTTTATCGCTCAGAG-3’ (Forward primer) and 5’-TGTCAGAGTACGGCTCCTG-3’ (Reverse primers). Oligonucleotide primers for GAPDH were 5’- GGCCTCCAAGGAGTAAGACC-3’ (forward primer) and 5’-CAAGGGGTCTACATGGCAAC-3’ (reverse primers). All ampliﬁcations and detections were carried out in the Applied Biosystems Prism 7900 system (Applied Biosystems, Foster City, CA) using the ExScript Sybr green QPCR kit (TaKaRa) and the following program: 95°C for 10 s, one cycle; 95°C for 5 s, 62°C for 31 s, 40 cycles; followed by a 30-min melting curve collection to verify the primer dimers. Statistical analysis was performed using the 2^-△△CT^ relative quantiﬁcation method.

### Immunoblotting analysis

To analyze RPS7 expression in cells, we prepared cell lysates at 75% of confluence using 500 µL of radioimmunoprecipitation assay buffer (RIPA, 25 mM Tris–HCl at pH 7.6, 150 mM NaCl, 1% Nonidet P-40, 1% sodium deoxycholate, and 0.1% sodium dodecyl sulfate). Protein concentrations of the lysates were determined with a Bio-Rad protein assay kit (Hercules, CA). Immunoblotting analyses were performed as described previously [22]. Antibodies against the following proteins were obtained from Santa Cruz Biotechnology: RPS7, BAX, Bcl-2, cyclin-dependent kinase (CDK) 2, CDK4, cyclin B1, cyclin D1, p21^waf1/cip1^, p27^cip/kip^, MMP2, MMP9, MMP13, AKT1, AKT2 and E-cadherin. Antibodies against the following proteins were from Cell Signaling Technology (Danvers, MA): β-actin, BAD, Bcl-XS, β-cantenin, MEK1/2, ERK1/2, JNK1/2, P38, P85α, P110α, pMEK1/2 (Ser217/221), pERK1/2 (Thr202/Tyr204), pJNK1/2(Thr183/Tyr185) and pP38 (Thr180/Tyr182). The secondary antibodies were F(ab)2 fragment of donkey anti-mouse immunoglobulin (product NA931) or of donkey anti-rabbit immunoglobulin (product NA9340) linked to horseradish peroxidase from Amersham Biosciences (Little Chalfont, Buckinghamshire,UK). Immunoblotting reagents were from an electrochemiluminescence kit (Amersham Biosciences). 

### Xenograft tumors in nude mice

All mouse experiments were performed following the institutional guidelines approved by the Institutional Animal Care and Use Committee of Fudan University Shanghai Cancer Center. Xenograft tumors were either subcutaneously or intraperitoneally generated to monitor tumor growth or metastasis of SKOV3-shRPS7 and OVCA433-shRPS cells and their controls. 4- to 6-week-old BALB/c athymic nude mice (Department of Laboratory Animal, Fudan University) were used for the experiment and kept in a pathogen-free environment. For the subcutaneous injection group, 5 × 10^6^ cells were used for each injection, and each mouse received two injections in bilateral flanks. Total five mice were used for each cell line. The date on which the first grossly visible tumor appeared for subcutaneous injection was recorded, and the tumor size was measured every 3 days. Two-dimensional measurements were taken with an electronic caliper, and tumor volume was calculated with the use of the following formula: tumor volume (in mm^3^) = a × b^2^ × 0.52 [23], where a is the longest diameter, b is the shortest diameter, and 0.52 is a constant to calculate the volume of an ellipsoid. When a tumor reached 1.0 cm in diameter, the mouse was killed by exposure to 5% carbon monoxide. Four tumors per cell line were excised, fixed in 10% formalin overnight, and subjected to routine histological examination by investigators who were blinded to the tumor status.

For the intraperitoneal injection group, eight mice were used for each cell line and each mouse received one injection of 5 × 10^6^ cells. Mice were observed for lethargy, poor appetite, and abdominal enlargement, and sacrificed timely before natural death occurred. Tumor nodules were counted, weighed, and measured for their numbers, weights, and volumes. Animal assays were repeated twice. 

### Immunohistochemical staining and analysis

Samples from 20 xenograft mouse tumors were used for immunohistochemical staining of E-cadherin and β-catenin expression. Antibodies used to detect E-cadherin and β-cantenin were from Santa Cruz Biotechnology (Santa Cruz, CA). The paraffin-embedded sections were pre-treated and stained with antibodies by using the previously reported method [20,24]. The secondary antibodies against mouse or rabbit IgG were supplied in an IHC kit (#CW2069) from Beijing CoWin Bioscience Co. Ltd (Beijing, China).

#### Statistical analysis

All data were analyzed by the Student *t* test. *P < 0.05* was considered statistically significant.

## Results

### Knockdown of RPS7 increases cell proliferation

**Figure 1:**
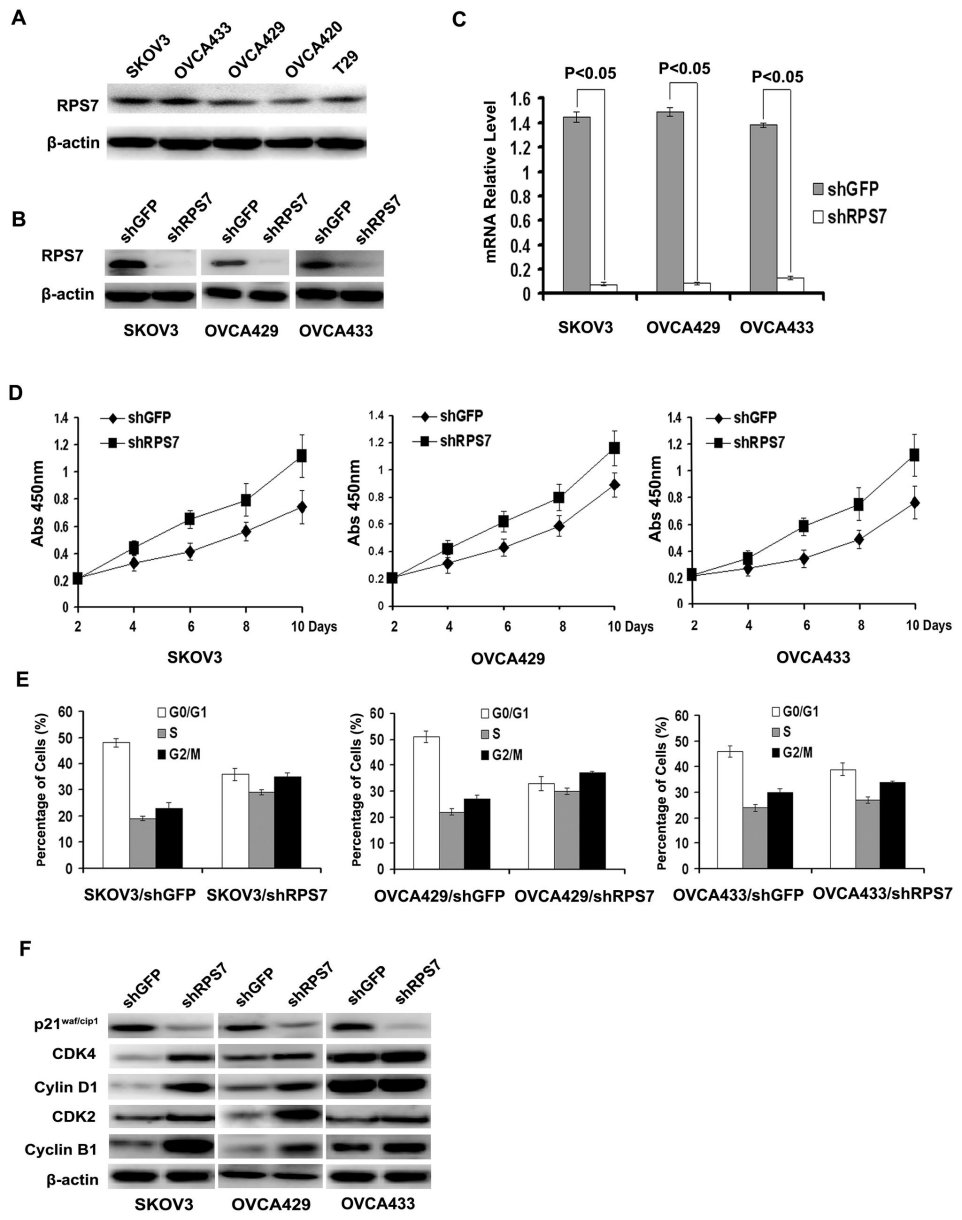
Expression of RPS7 in ovarian cancer cells and immortalized ovarian surface epithelial cells and effects of RPS7 on cell proliferation and cell cycle. **A**, Detection of RPS7 expression by Western blotting in SKOV3, OVCA433, OVCA429, OVCA420 and T29 cells. **B**-**C**, Analyses of RPS7 by Western blotting (B) and realtime-PCR (C) in cells expressing shGFP or shRPS7. **D**, Detection of cell proliferation by CCK8 (*P < 0.05*). Error bars = 95% confidence intervals (CIs). **E**, Quantitative analysis of cell cycle distribution. Data from three independent experiments were analyzed (*P < 0.05*). Error bars = 95% CIs. **F**, Immunoblotting analysis of cell cycle regulatory proteins. β-actin was used as the loading control.

To investigate the function of RPS7 in human ovarian cancer, we first detected the expression level of RPS7 in four human ovarian cancer cell lines and one immortalized human ovarian surface epithelial cell line (T29). We found that the expression of RPS7 was higher in SKOV3, OVCA429 and OVCA433 cells than in OVCA420 and T29 cells (Figure **1A**). Thus, we introduced retroviruses carrying shRNA against RPS7 into SKOV3, OVCA429 and OVCA433 cells to generate SKOV3-shRPS7, OVCA429-shRPS7 and OVCA433-shRPS7 cells (corresponding control cells were infected with retroviruses expressing shGFP). As detected by Western blotting and realtime PCR, respectively, both the mRNA level and the protein level of RPS7 were decreased in cells treated with RPS7 shRNA compared with in controls (Figure **1B-C**). We next evaluated cell proliferation rates of the cell lines by CCK8 assay. The results showed that cells expressing shRNA had higher level of proliferation than the corresponding control cells expressing shGFP (Figure **1D**).

### Down-regulation of RPS7 promotes cell cycle progression

Previous studies have shown that RPS7 may accelerate the G2-M phase transition of cell cycle during the early development of zebrafish embryo [13]. We found that the cell population was decreased at the G0-G1 phase but increased at both S and G2-M phases in three cell lines treated with RPS7 shRNA compared with in control cells (Figure **1E**). To explore the potential mechanism, we analyzed major proteins associated with cell cycle progression by Western blotting. The results in Figure **1F** showed that p21^Cip1/Waf1^, an essential suppressor involved in the G1-S cell cycle transition, was remarkably decreased in SKOV3, OVCA429, and OVCA433 cells after RPS7 was knocked down compared with in control cells (Figure **1F**). Cyclin-dependant kinase 4 (Cdk 4) was markedly increased in SKOV3-shRPS7 and OVCA429-shRPS7 cells, but was slightly increased in OVCA433-shRPS7 cells compared with in control cells expressing shGFP. Cyclin D1, a Cdk4 partner protein, was not altered in OVCA433-shRPS7, but was increased in OVCA429-shRPS7 and SKOV3-shRPS7 cells (Figure **1F**), compared with in their control cells. The S-phase regulatory protein Cdk2 was markedly increased in all cells whose expression of RPS7 was silenced, compared with in their control cells. Meanwhile, we found that cyclin B1, a G2-M transition-promoting protein, was increased in SKOV3, OVCA429 and OVCA433 cells after RPS7 was silenced. These data suggested that RPS7 promotes ovarian cancer cell cycle progression in the G1-S and G2-M transitions, possibly through suppression of p21^Cip1/Waf1^. Other factors, such as Cdk4 and cyclin B1, may also be involved in RPS7-associated cell cycle regulation.

### Knockdown of RPS7 attenuates cellular apoptosis in ovarian cancer cells

**Figure 2:**
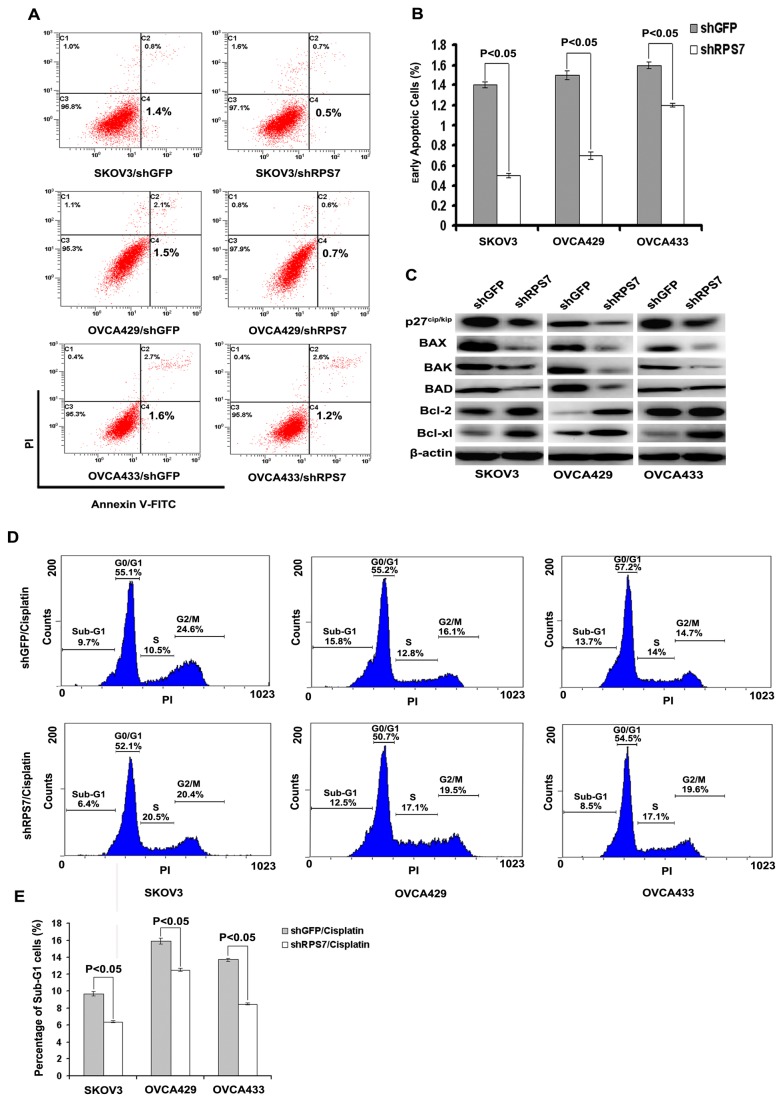
Examination of cell apoptosis associated with RPS7. **A**, Apoptosis detected by flow cytometry. **B**, Quantitative analysis of apoptotic cells (*P < 0.05*). Error bars = 95% CIs. **C**, Immunoblotting analysis of apoptosis-associated proteins. β-actin was used as the loading control. **D**, Cisplatin-induced apoptosis indicated as sub-G1 population detected by flow cytometry. **E**, Quantitative analysis of sub-G1 cells (*P < 0.05*). Error bars = 95% CIs.

We investigated the effect of RPS7 expression on cellular apoptosis using annexin v fluorescence apoptosis assay. Although silencing of RPS7 expression slightly reduced cell apoptosis (Figure **2A-B**), a decreased expression of the pro-apoptotic proteins p21^waf1/cip1^, p27^cip/kip^, BAX, BAK and BAD, and an enhanced expression of the anti-apoptotic proteins Bcl-2 and Bcl-xl were detected in shRPS7-treated cells, compared with in control cells (Figure **1F** and Figure **2C**). These data suggested that RPS7 might contribute to cellular apoptosis. 

To validate the above results, we treated SKOV3-shRPS7, OVCA429-shRNA, OVCA433-shRPS7 cells, and their control cells with cisplatin and used flow cytometry to detect cell apoptosis indicated as the percentage of sub-G1 cells with PI staining (chromosomes < 2n). We found that, with the stimulation of cisplatin at 2.5 µg/mL, the percentage of sub-G1 cells was lower in SKOV3-shRPS7 (6.4%), OVCA429-shRNA (12.5%) or OVCA433-shRPS7 (8.5%) cells than that in SKOV3-shGFP (9.7%), OVCA429-shGFP (15.8%), or OVCA433-shGFP (13.7%) cells (Figure **2D-E**) (*P* < 0.05). However, without cisplatin treatment, the percentage of sub-G1 cells was much lower in both shRNA-treated cells and their control cells and had no statistical significance between shRNA-treated cells and their control cells (Figure S1) (*P* > 0.05). These results suggested that knock down of RPS7 moderately attenuated cancer cells apoptosis and chemo-sensitivity to cisplatin treatment. 

### RPS7 regulates PI3K/AKT and MAPK signal networks

**Figure 3:**
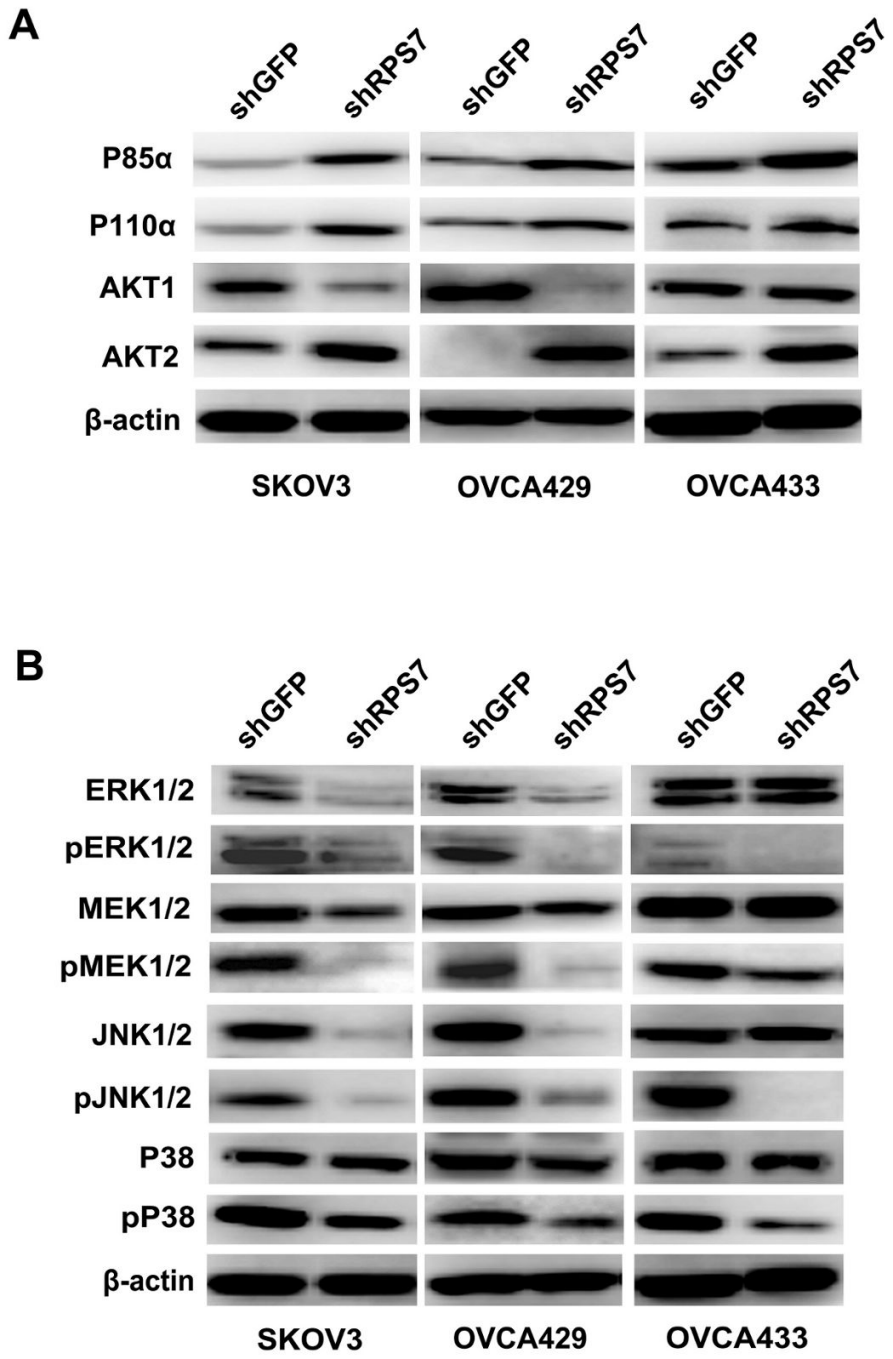
Detection of PI3K/AKT and MAPK signal pathways by Western blotting. **A**, Immunoblotting analysis of molecules associated with PI3K/AKT signal pathway. β-actin was used as the loading control. **B**, Immunoblotting analysis of molecules associated with MAPK signal pathway. β-actin was used as the loading control.

Since PI3K/AKT and MAPK are two important intracellular pathways involved in cell proliferation, cell cycle, and apoptosis, we detected some important members in PI3K/AKT and MAPK signal networks by Western blotting. We found that the levels of P85α and P110α, two main subunits of PI3K, were increased in SKOV3-shRPS7, OVCA429-shRPS7 or OVCA433-shRPS7 cells (Figure **3A**), compared within their controls. AKT1 was decreased in SKOV3 and OVCA429, but not changed in OVCA433 after RPS7 was silenced (Figure **3A**), whereas AKT2 was increased in all three cell lines in which RSP7 was silenced (Figure **3A**), compared with in cells expressing shGFP. 

ERK 1/2, MEK1/2 and JNK1/2 were reduced in SKOV3-shRPS7 and OVCA429-shRPS7, but not in OVCA433-shRPS7 (Figure **3B**), and the phosphorylation of MEK1/2 (Ser217/221), ERK1/2 ( Thr202/Tyr204), and JNK1/2(Thr183/Tyr185) was reduced in SKOV3-shRPS7, OVCA429-shRPS7 and OVCA433-shRPS7 cells, compared with in their controls (Figure **3B**). The phosphorylation of P38 (Thr180/Tyr182) was reduced in all three ovarian cancer cell lines after RPS7 was silenced, but P38 was not changed in SKOV3-shRPS7, OVCA429-shRPS7 and OVCA433-shRPS7 cells (Figure **3B**). Thus, based on the above results, we infer that RPS7 may regulate cell proliferation, cell cycle, and cell apoptosis through PI3K/AKT and MAPK signaling.

### Knockdown of RPS7 promotes cell invasion and migration

**Figure 4:**
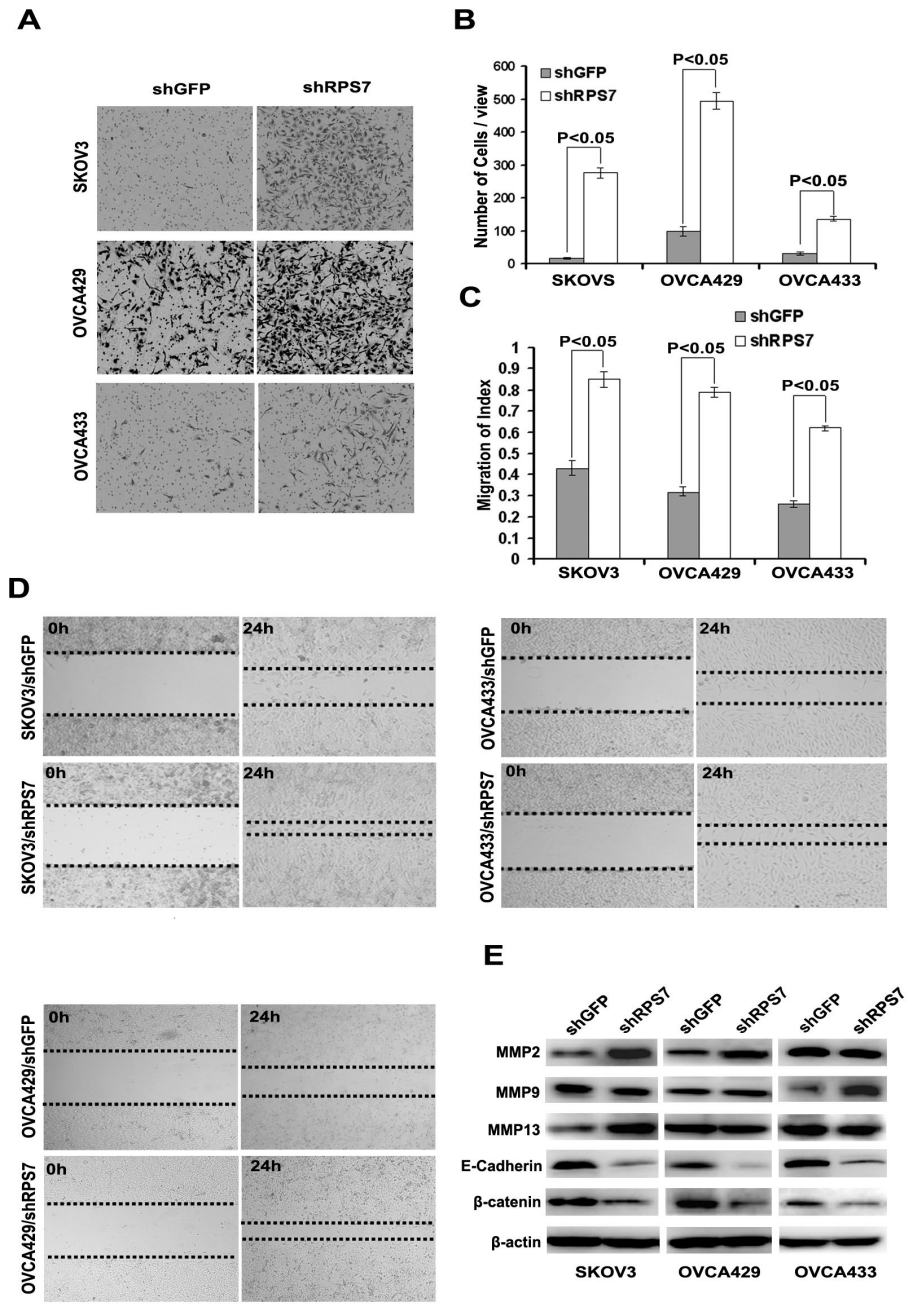
Influence of cell invasion and migration by RPS7. **A**, Detection of cell invasion by using a high throughput screening multi-well insert 24-well two-chamber plates. **B**, Quantitative analysis of migration cells (*P < 0.05*). Error bars = 95% CIs. **C**, Quantitative analysis of migration speed with migration index (*P < 0.05*). Error bars = 95% CIs. **D**, Detection of migration speed by scratch assay. **E**, Immunoblotting analysis of metastasis-associated proteins. β-actin was used as the loading control.

To investigate the function of RPS7 in invasion and migration, we performed assays using a high throughput transwell assay. We found that more SKOV3-shRPS7, OVCA429-shRPS7 and OVCA433-shRPS7 cells invaded through the membrane in the bottom chamber than their control cells (Figure **4A-B**). We also detected migration speed by scratch assay and found that the migration speed of SKOV3-shRPS7, OVCA429-shRPS7 or OVCA433-shRPS7 cells was increased after 24h culture compared with control cells (Figure **3C-D**). Further, we determined the expression of migration-related proteins, including members of matrix metalloproteinase family (MMP), E-cadherin, and β-catenin. Compared with in control cells, the expression level of MMP2 was up-regulated in SKOV3-shRPS7 cells and OVCA429-shRPS7, but was not changed in OVCA433-shRPS7 cells (Figure **4E**). MMP9 was increased in OVCA433-shRPS7 cells, but not in SKOV3-shRPS7 and OVCA429-shRPS7 cells (Figure **4E**). MMP13 was increased in SKOV3-shRPS7 cells, but not in OVCA429-shRPS7 and OVCA433-shRPS7 cells (Figure **4E**). Both E-cadherin and β-catenin were decreased in all ovarian cancer cell lines whose RPS7 was silenced (Figure **4E**). Based on these results, RPS7 appears to influence ovarian cancer cell invasion and migration possibly through regulation of MMP2, E-cadherin, and β-catenin.

### RPS7 is involved in ovarian tumorigenesis and metastasis

**Figure 5:**
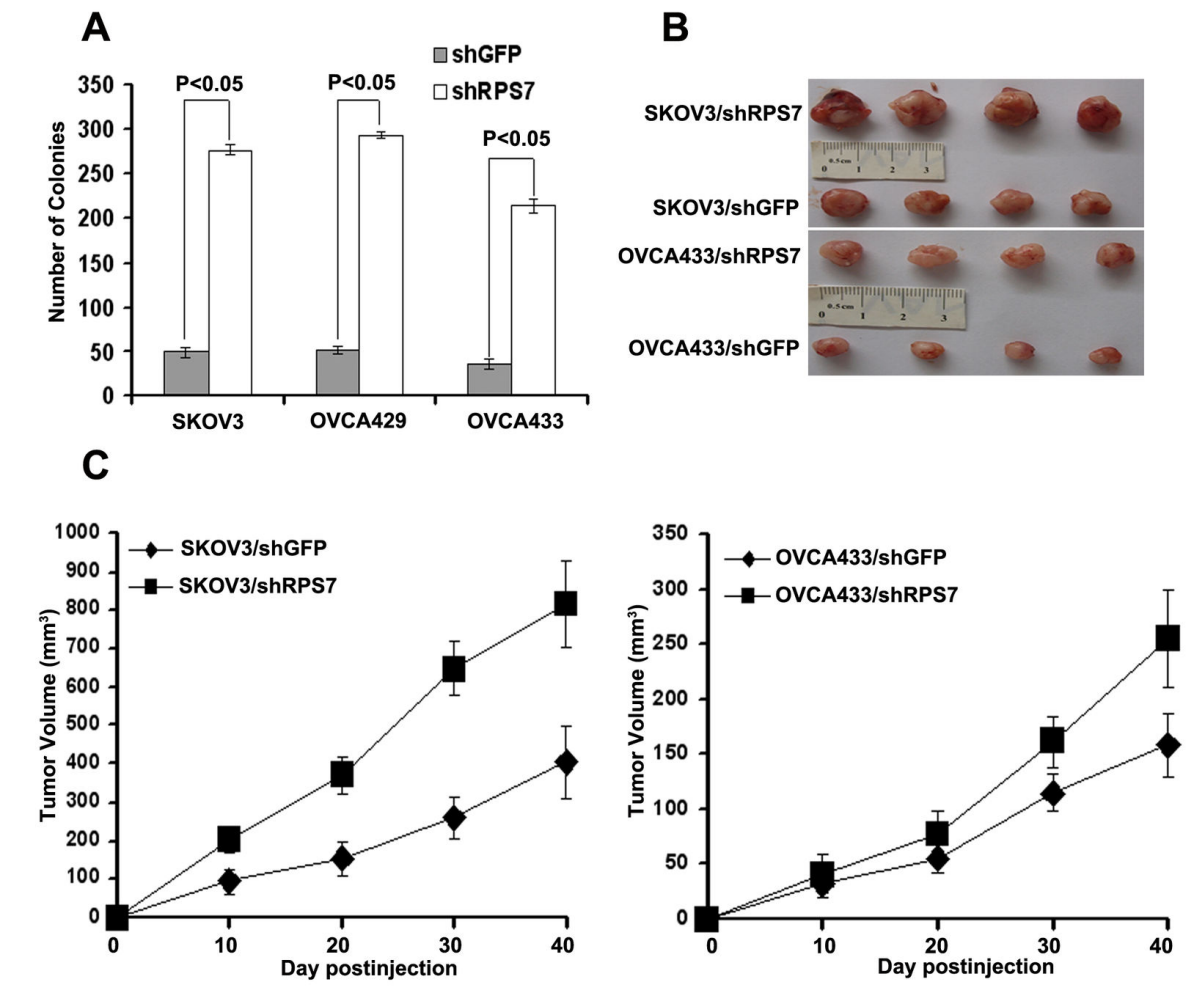
Xenograft tumor burden in mice with or without silencing of RPS7. **A**, In vitro tumorigenicity tested by soft agar assay (*P < 0.05*). Error bars = 95% CIs. **B**, In vivo tumorigenesis examined by animal assay. **C**, Subcutaneous tumor growth from mice injected with cells expressing either shGFP or shRPS7 (*P < 0.05*). Error bars = 95% CIs.

To determine the roles of RPS7 in ovarian tumorigenesis, we first performed the anchorage-independent growth assay and found that SKOV3-shRPS7, OVCA429-shRPS7 and OVCA433-shRPS7 cells generated a greater number of colonies than their control cells (P < 0.05) (Figure **5A**). To test the in vivo effect of RPS7 on tumorigenesis, SKOV3-shRPS7 or OVCA433-shRPS7 cells and their corresponding controls were subcutaneously injected into nude mice. As shown in Figure **5B-C**, the tumors from nude mice injected with SKOV3-shRPS7 or OVCA433-shRPS7 cells grew faster and to a larger volume than those derived from control cells (P < 0.05). 

**Figure 6:**
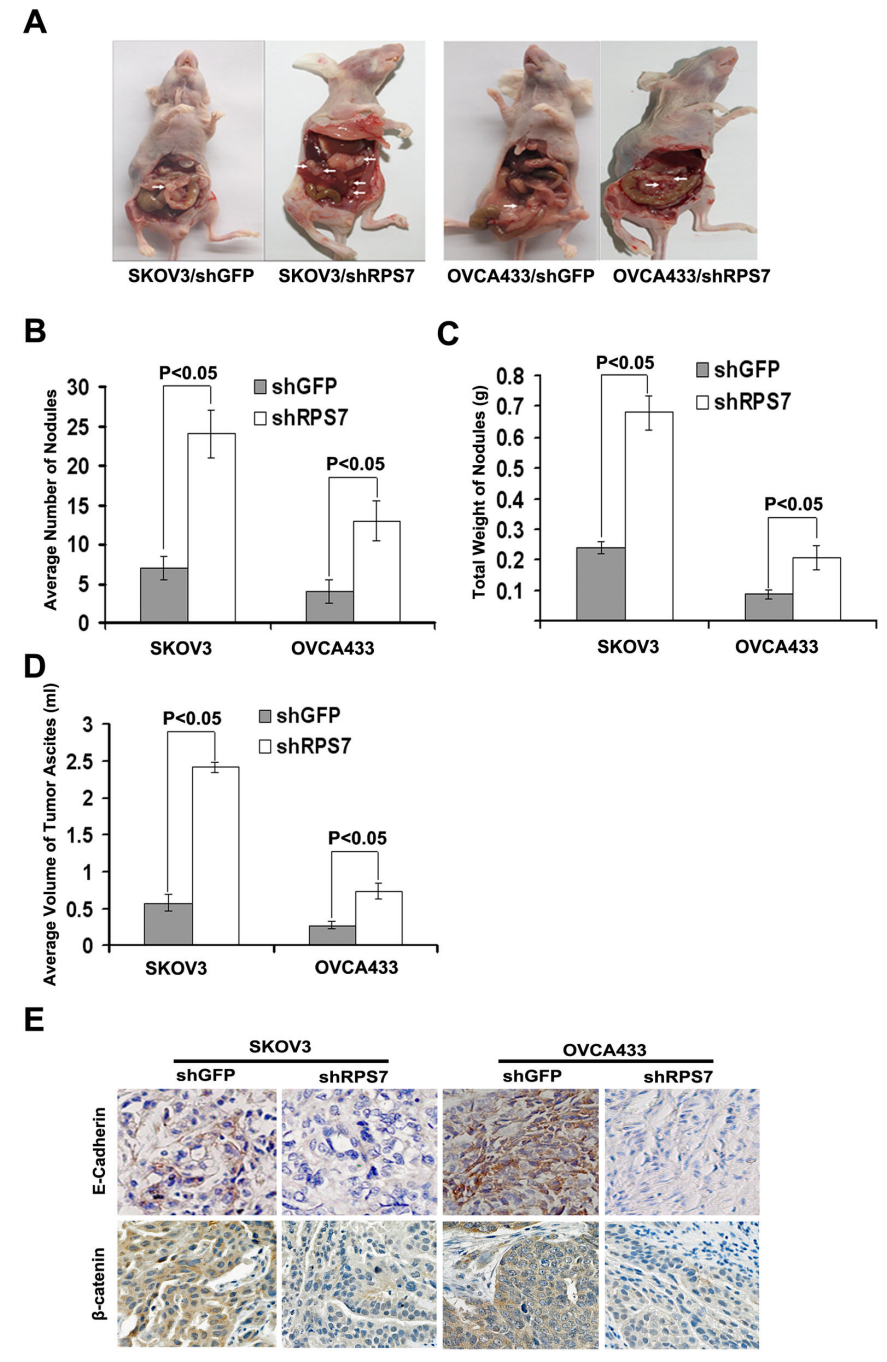
Analysis of intraperitoneally generated xenograft tumors and tissues. **A**, Dissection of xenograft tumor. **B**, Quantitative analysis of the number of nodules in nude mice (*P < 0.05*). Error bars = 95% CIs. **C**, Quantitative analysis of the weight of nodules in nude mice (*P < 0.05*). Error bars = 95% CIs. **D**, Quantitative analysis of the volume of tumor ascites in nude mice (*P < 0.05*). Error bars = 95% CIs. **E**, Immunohistochemical staining of E-cadherin and β-catenin in xenograft tumor tissues. Tissues were stained with mouse anti- E-cadherin andβ-catenin antibodies and visualized with donkey anti-mouse secondary antibody (Magnification×200).

To further explore the function of RPS7 in cancer cell metastasis, SKOV3-shRPS7 or OVCA433-shRPS7 cells and their corresponding controls were intraperitoneally injected into nude mice. The results showed that silencing of RPS7 increased the number and weight of nodules (Figure **6B**) and tumor ascites (Figure **6C-E**) in mice injected with cells treated with shRPS7 compared with mice injected with cells expressing shGFP (P < 0.05).

The analysis of tumors from nude mice by immunohistochemical staining showed that the expression of both E-cadherin and β-catenin in tumor tissue from nude mice injected with SKOV3-shRPS7 or OVCA433-shRPS7 cells were reduced compared with those in tumor tissues derived from mice injected with corresponding control cells (Figure **6A**). These results suggested that RSP7 might function to suppress tumor growth and metastasis during the development of ovarian cancer, which is consistent with the data that RSP7 inhibits cell invasion and migration (Figure **4**).

## Discussion

In this study, we found that the aberrant expression of RPS7 might be associated with the development of ovarian cancer. Silencing of RPS7 promoted cell proliferation through dysregulation of p21^waf1/cip1^, CDK2, CDK4, Cyclin B1, and Cyclin D1. Moreover, knockdown of RPS7 down-regulated the expression of pro-apoptotic factors such as p27^cip/kip^, BAX, BAK, and BAD, but up-regulated the expression of anti-apoptotic factors, including Bcl-2 and Bcl-xl, which resulted in attenuated apoptosis. Subsequently, we found that silencing of RPS7 enhanced cell migration and invasion, which is consistent to the regulatory functions of RPS7 in cell cycle and apoptosis. Further studies revealed that RPS7 might control the PI3K/AKT and MAPK signal networks to inhibit ovarian tumorigenesis and metastasis. 

Over the past decades, ribosomal proteins (RPs) have been found to play important roles in the development of many cancers, including colon carcinoma, breast cancer, esophageal cancer, and hepatocellular carcinoma [25-27]. Extensive evidences on RPs’ tumorigenic functions have been documented these years. Wu et al. found that overexpression of RPL6 in human gastric tissues predicted poor prognosis. Up-regulation of RPL6 accelerated cell growth and enhanced in vitro colony formation of gastric cancer cells, while down-regulation of RPL6 could suppress cell cycle progression, at least partially through down-regulating cyclin E, indicating that RPL6 might be used as a novel therapeutic target for gastric cancer [4]. Llanos et al. found that the ectopic overexpression of RPL37 attenuated the DNA damage response mediated by p53, which supports the notion that the DNA damage-induced proteasome degradation of RPL37 constitutes a mechanistic link between DNA damage and ribosomal stress pathway [28]. Kobayashi et al. confirmed that knockdown of RPL13 using small interfering RNA ( siRNA) resulted in drastic attenuation of gastrointestinal cancer cell growth with significant G1 and G2/M arrest of cell cycle [5]. Further, they found that RPL13 siRNA significantly enhanced the cellular sensitivity to certain DNA damaging agents and, concordantly, RPL13-overexpressing cells displayed greater chemoresistance than the parental cells, suggesting that an inverse correlation exists between RPL13 expression and chemosensitivity [5]. However, Chien et al. reported that RPS19 in colorectal cancer (CRC) possibly promoted cellular apoptosis through BAX/p53 pathway, and that the level of fecal RPS19 might be used as a favorable prognostic factor for CRC patients [29]. Thus, the function of RPs involved in cell proliferation, cell cycle, apoptosis and tumorigenesis varies depending on tissues and cell types studied in literatures. Our results demonstrate that RPS is abnormally expressed in ovarian cancer cell lines and tissues and plays an important role in ovarian tumorigenesis and chemo-sensitivity. However, the clinical significance of RPS7 in ovarian cancer remains to be investigated in future.

In conclusion, we demonstrate that RPS7 inhibits ovarian tumor growth and metastasis through regulation of the PI3K/AKT and MAPK signal pathways. 

## Supporting Information

Figure S1 **Quantitative analysis of sub-G1 cells in cell lines without treatment of cisplatin** (**P > 0.05)**.
Error bars = 95% CIs.
(PDF)

## References

[1] Morgan RJ, Alvarez RD, Armstrong DK, Burger RA, Castells M et al. (2012) Ovarian Cancer, Version 3.2012. J Natl Compr Canc Netw 10: 1339-1349. 23138163 10.6004/jnccn.2012.0140

[2] Seong KM, Jung SO, Kim HD, Kim HJ, Jung YJ et al. (2012) Yeast ribosomal protein S3 possesses a beta-lyase activity on damaged DNA. FEBS Lett 586: 356-361. doi:10.1016/j.febslet.2011.12.030. PubMed: 22245673. 22245673

[3] Kobayashi Y, Kawakami K, Ohbayashi M, Kohyama N, Yamamoto T (2010) Ribosomal protein L3 mediated the transport of digoxin in Xenopus laevis oocyte. J Toxicol Sci 35: 827-834. doi:10.2131/jts.35.827. PubMed: 21139332. 21139332

[4] Wu Q, Gou Y, Wang Q, Jin H, Cui L et al. (2011) Down-regulation of RPL6 by siRNA inhibits proliferation and cell cycle progression of human gastric cancer cell lines. PLOS ONE 6: e26401. doi:10.1371/journal.pone.0026401. PubMed: 22043320. 22043320 PMC3197136

[5] Kobayashi T, Sasaki Y, Oshima Y, Yamamoto H, Mita H et al. (2006) Activation of the ribosomal protein L13 gene in human gastrointestinal cancer. Int J Mol Med 18: 161-170. PubMed: 16786168. 16786168

[6] Kondoh N, Schweinfest CW, Henderson KW, Papas TS (1992) Differential expression of S19 ribosomal protein, laminin-binding protein, and human lymphocyte antigen class I messenger RNAs associated with colon carcinoma progression and differentiation. Cancer Res 52: 791-796. PubMed: 1339304. 1339304

[7] Li C, Ge M, Yin Y, Luo M, Chen D (2012) Silencing expression of ribosomal protein L26 and L29 by RNA interfering inhibits proliferation of human pancreatic cancer PANC-1 cells. Mol Cell Biochem 370: 127-139. doi:10.1007/s11010-012-1404-x. PubMed: 22868929. 22868929

[8] Bee A, Brewer D, Beesley C, Dodson A, Forootan S et al. (2011) siRNA knockdown of ribosomal protein gene RPL19 abrogates the aggressive phenotype of human prostate cancer. PLOS ONE 6: e22672. doi:10.1371/journal.pone.0022672. PubMed: 21799931. 21799931 PMC3142177

[9] Gou Y, Shi Y, Zhang Y, Nie Y, Wang J et al. (2010) Ribosomal protein L6 promotes growth and cell cycle progression through up-regulating cyclin E in gastric cancer cells. Biochem Biophys Res Commun 393: 788-793. doi:10.1016/j.bbrc.2010.02.083. PubMed: 20171175. 20171175

[10] Wang Q, Yang C, Zhou J, Wang X, Wu M et al. (2001) Cloning and characterization of full-length human ribosomal protein L15 cDNA which was overexpressed in esophageal cancer. Gene 263: 205-209. doi:10.1016/S0378-1119(00)00570-9. PubMed: 11223259. 11223259

[11] Wu LM, Wang SY, Wang SX, Huang YM, Li JG (2010) [Proliferation promotion and apoptotic inhibition effects of ribosomal protein RPL36A small interference RNA on U937 cells]. Zhongguo Shi Yan Xue Ye Xue Za Zhi 18: 344-349. PubMed: 20416165. 20416165

[12] Wu CT, Lin TY, Hsu HY, Sheu F, Ho CM et al. (2011) Ling Zhi-8 mediates p53-dependent growth arrest of lung cancer cells proliferation via the ribosomal protein S7-MDM2-p53 pathway. Carcinogenesis 32: 1890-1896. doi:10.1093/carcin/bgr221. PubMed: 21983128. 21983128

[13] Duan J, Ba Q, Wang Z, Hao M, Li X et al. (2011) Knockdown of ribosomal protein S7 causes developmental abnormalities via p53 dependent and independent pathways in zebrafish. Int J Biochem Cell Biol 43: 1218-1227. doi:10.1016/j.biocel.2011.04.015. PubMed: 21550419. 21550419

[14] Zhu Y, Poyurovsky MV, Li Y, Biderman L, Stahl J et al. (2009) Ribosomal protein S7 is both a regulator and a substrate of MDM2. Mol Cell 35: 316-326. doi:10.1016/j.molcel.2009.07.014. PubMed: 19683495. 19683495 PMC2896961

[15] Chen D, Zhang Z, Li M, Wang W, Li Y et al. (2007) Ribosomal protein S7 as a novel modulator of p53-MDM2 interaction: binding to MDM2, stabilization of p53 protein, and activation of p53 function. Oncogene 26: 5029-5037. doi:10.1038/sj.onc.1210327. PubMed: 17310983. 17310983

[16] Auersperg N, Siemens CH, Myrdal SE (1984) Human ovarian surface epithelium in primary culture. In Vitro 20: 743-755. doi:10.1007/BF02618290. PubMed: 6083974. 6083974

[17] Liu J, Yang G, Thompson-Lanza JA, Glassman A, Hayes K et al. (2004) A genetically defined model for human ovarian cancer. Cancer Res 64: 1655-1663. doi:10.1158/0008-5472.CAN-03-3380. PubMed: 14996724. 14996724

[18] Riccardi C, Nicoletti I (2006) Analysis of apoptosis by propidium iodide staining and flow cytometry. Nat Protoc ;1(3): 1458-1461. doi:10.1038/nprot.2006.238. PubMed: 17406435. 17406435

[19] Yang G, Chang B, Yang F, Guo X, Cai KQ et al. (2010) Aurora kinase A promotes ovarian tumorigenesis through dysregulation of the cell cycle and suppression of BRCA2. Clin Cancer Res 16: 3171-3181. doi:10.1158/1078-0432.CCR-09-3171. PubMed: 20423983. 20423983 PMC2930838

[20] Yang G, Rosen DG, Zhang Z, Bast RC Jr., Mills GB et al. (2006) The chemokine growth-regulated oncogene 1 (Gro-1) links RAS signaling to the senescence of stromal fibroblasts and ovarian tumorigenesis. Proc Natl Acad Sci U S A 103: 16472-16477. doi:10.1073/pnas.0605752103. PubMed: 17060621. 17060621 PMC1637606

[21] Yang G, Rosen DG, Liu G, Yang F, Guo X et al. (2010) CXCR2 promotes ovarian cancer growth through dysregulated cell cycle, diminished apoptosis, and enhanced angiogenesis. Clin Cancer Res 16: 3875-3886. doi:10.1158/1078-0432.CCR-10-0483. PubMed: 20505188. 20505188 PMC2930833

[22] Yang G, Thompson JA, Fang B, Liu J (2003) Silencing of H-ras gene expression by retrovirus-mediated siRNA decreases transformation efficiency and tumorgrowth in a model of human ovarian cancer. Oncogene 22: 5694-5701. doi:10.1038/sj.onc.1206858. PubMed: 12944918. 12944918

[23] Liu G, Yang G, Chang B, Mercado-Uribe I, Huang M et al. (2010) Stanniocalcin 1 and ovarian tumorigenesis. J Natl Cancer Inst 102: 812-827. doi:10.1093/jnci/djq127. PubMed: 20484106. 20484106 PMC2879417

[24] Yang G, Xiao X, Rosen DG, Cheng X, Wu X et al. (2011) The biphasic role of NF-kappaB in progression and chemoresistance of ovarian cancer. Clin Cancer Res 17: 2181-2194. doi:10.1158/1078-0432.CCR-10-3265. PubMed: 21339307. 21339307 PMC3152795

[25] Lindström MS, Nistér M (2010) Silencing of ribosomal protein S9 elicits a multitude of cellular responses inhibiting the growth of cancer cells subsequent to p53 activation. PLOS ONE 5: e9578. doi:10.1371/journal.pone.0009578. PubMed: 20221446. 20221446 PMC2833189

[26] Guo X, Shi Y, Gou Y, Li J, Han S et al. (2011) Human ribosomal protein S13 promotes gastric cancer growth through down-regulating p27(Kip1). J Cell Mol Med 15: 296-306. doi:10.1111/j.1582-4934.2009.00969.x. PubMed: 19912438. 19912438 PMC3822796

[27] Bee A, Ke Y, Forootan S, Lin K, Beesley C et al. (2006) Ribosomal protein l19 is a prognostic marker for human prostate cancer. Clin Cancer Res 12: 2061-2065. doi:10.1158/1078-0432.CCR-05-2445. PubMed: 16609016. 16609016

[28] Llanos S, Serrano M (2010) Depletion of ribosomal protein L37 occurs in response to DNA damage and activates p53 through the L11/MDM2 pathway. Cell Cycle 9: 4005-4012. doi:10.4161/cc.9.19.13299. PubMed: 20935493. 20935493 PMC3615335

[29] Chien CC, Tu TC, Huang CJ, Yang SH, Lee CL (2012) Lowly expressed ribosomal protein s19 in the feces of patients with colorectal cancer. ISRN. Gastroenterologist 2012: 394545. 10.5402/2012/394545 PMC3261477 22272377

