# Supplementary figures and images for: Small Ribosomal Protein Subunit S7 Suppresses Ovarian Tumorigenesis through Regulation of the PI3K/AKT and MAPK Pathways
Source: PLoS One. 2013 Nov 11;8(11):e79117. doi: 10.1371/journal.pone.0079117 (PMC3823983; doi:10.1371/journal.pone.0079117)

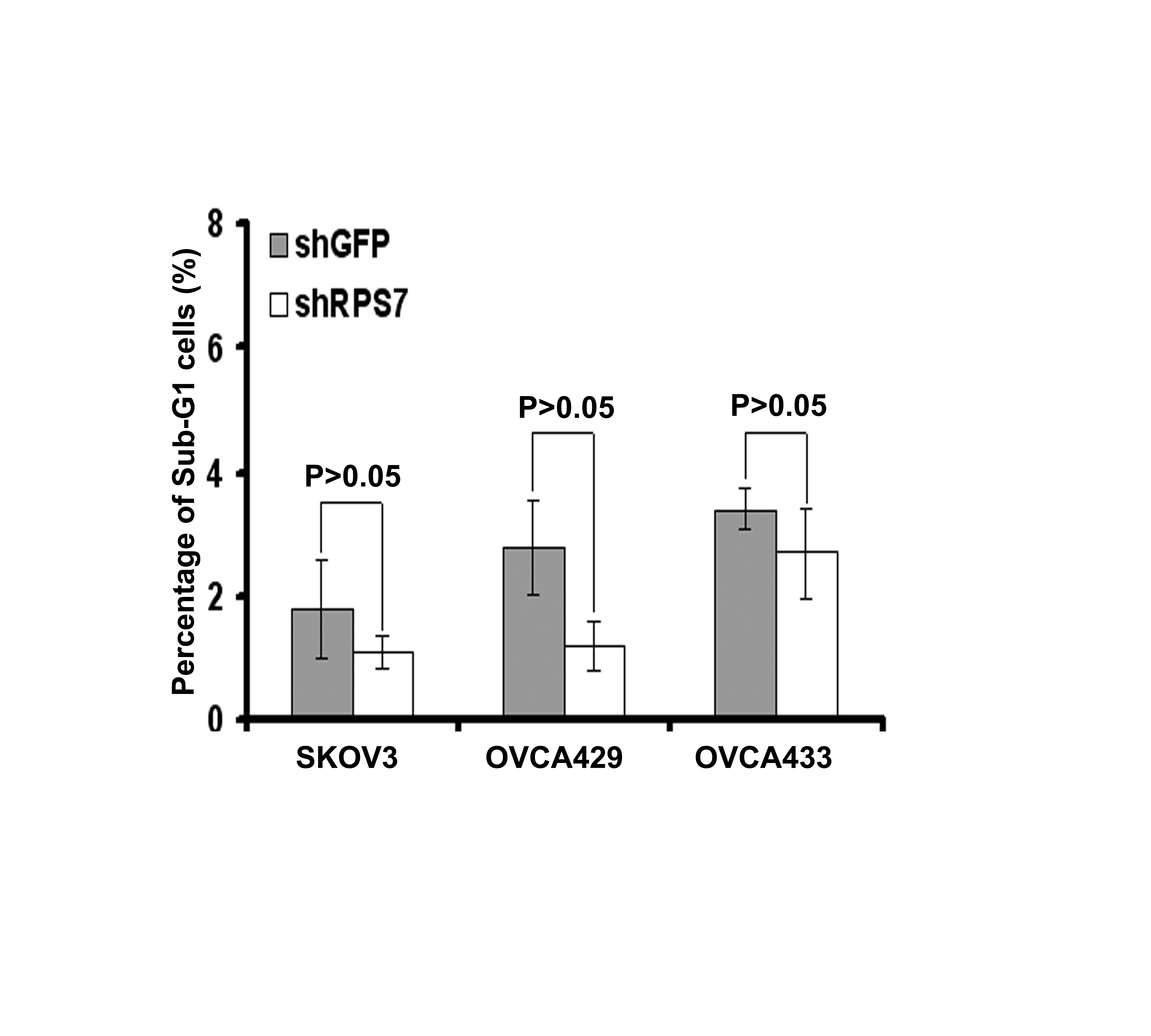

Supplement: Figure S1 — Quantitative analysis of sub-G1 cells in cell lines without treatment of cisplatin (P > 0.05). Error bars = 95% CIs. (PDF) [file pone.0079117.s001.pdf]
